# *Aedes aegypti* and dengue: insights into transmission dynamics and viral lifecycle

**DOI:** 10.1017/S0950268825100320

**Published:** 2025-08-01

**Authors:** Ebrahim Abbasi

**Affiliations:** 1 Research Center for Health Sciences, Institute of Health, Shiraz University of Medical Sciences, Shiraz, Iran; 2 Department of Medical Entomology and Vector Control, School of Health, Shiraz University of Medical Sciences, Shiraz, Iran

**Keywords:** *Aedes aegypti*, arboviral transmission, climate change, dengue virus, molecular interactions, vector competence

## Abstract

Dengue virus (DENV) remains a pressing global health challenge, primarily transmitted by *Aedes aegypti* mosquitoes. This review synthesizes current knowledge on the biological, environmental, and molecular factors influencing DENV transmission, drawing upon 120 peer-reviewed studies. The narrative analysis highlights the mosquito’s vector competence, shaped by genetic variability, midgut barriers, and immune responses. Environmental drivers particularly temperature, humidity, and urbanization emerge as critical determinants of transmission dynamics. A meta-analysis of 30 studies reveals a strong positive correlation (*r* = 0.85, *p* < 0.01) between temperature (25 °C–30 °C) and transmission efficiency. Proteomic studies further detail molecular interactions facilitating viral entry and replication. Although novel interventions such as Wolbachia-based biocontrol and genetic modification show promise, context-specific implementation remains challenging, especially in low-resource settings. Key research gaps include the impact of climate change, co-infections with other arboviruses, and the long-term efficacy of vector control innovations. Prioritizing interdisciplinary approaches and adapting strategies to local contexts are vital to reducing the dengue burden and informing future public health responses.

## Graphical abstract

**Figure:**
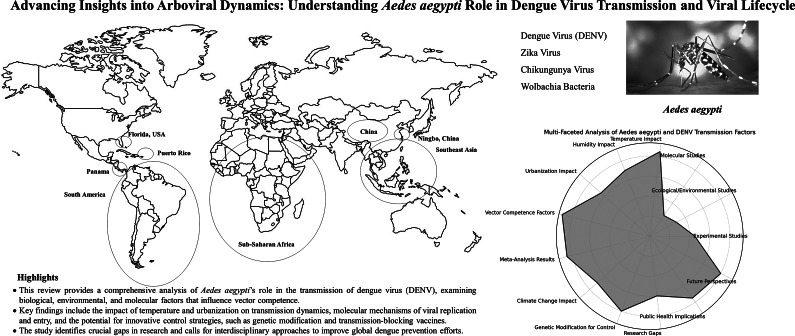

## Introduction

Arthropod-borne viruses (arboviruses), notably dengue virus (DENV), constitute a major global health threat, particularly in tropical and subtropical climates where environmental conditions favour mosquito proliferation. Dengue, an acute febrile illness caused by DENV and transmitted predominantly by *A. aegypti* mosquitoes, is currently among the most widespread mosquito-borne viral infections. According to the World Health Organization (WHO), nearly 50% of the global population resides in areas at risk for dengue transmission, with an estimated 100–400 million infections occurring annually. Despite ongoing efforts in vector control and public health interventions, dengue remains a significant source of morbidity and mortality, straining healthcare infrastructure and impacting socio-economic stability in endemic regions [1–3].

A comprehensive understanding of *A. aegypti*’s role in the transmission dynamics of DENV is fundamental to the development of targeted and sustainable control strategies. As a highly anthropophilic and ecologically adaptable mosquito species, *A. aegypti* possesses a set of biological and behavioural characteristics that enhance its vectorial capacity. These include a strong preference for feeding on humans, a tendency to rest and breed in indoor environments, and a relatively short extrinsic incubation period (EIP), particularly at favourable ambient temperatures. Notably, the vector’s capacity to support DENV replication and systemic dissemination enables efficient viral transmission. The DENV transmission cycle is characterized by complex interactions between the virus, the mosquito vector, and the human host, with each component influencing the epidemiology and clinical manifestation of the disease [1, 4–8].

Investigations into the molecular and ecological determinants of *A. aegypti*’s vector competence have provided pivotal insights into the mechanisms underlying DENV evolution, adaptation, and sustained transmission. These studies underscore the significant role of environmental factors, particularly temperature and relative humidity, in modulating viral replication and transmission efficiency. Additionally, intrinsic factors such as genetic variation among mosquito populations and the vector’s innate immune responses critically influence its capacity to acquire, replicate, and transmit DENV. While substantial progress has been made in elucidating these host–pathogen interactions, numerous aspects of the complex virus–vector interface remain insufficiently characterized. This knowledge gap underscores the need for continued multidisciplinary research to unravel the intricate biological and ecological processes that drive arboviral emergence and persistence [9–12].

This review aims to synthesize current knowledge on the role of *A. aegypti* in DENV transmission and its implications for public health. By examining the biological, environmental, and molecular factors that underpin this relationship, we seek to illuminate potential avenues for innovative control strategies and contribute to a deeper understanding of arboviral transmission dynamics [13, 14].

In this review, a narrative methodology was intentionally selected instead of a systematic review due to the interdisciplinary nature of the subject matter. The transmission of DENV by *A. aegypti* involves complex biological, molecular, ecological, and environmental factors that span diverse research domains. A narrative review offers the flexibility to integrate and interpret findings across these varied fields, allowing for a more holistic and conceptual synthesis. Additionally, the heterogeneity of the existing literature in terms of study design, geographic context, and outcome measures presents challenges to applying a rigid systematic review framework. While not exhaustive in the manner of a systematic review, this narrative synthesis aims to comprehensively reflect major findings, emphasize emerging themes, and identify gaps to inform future research and control strategies [15–17].

## Materials and methods

### Study design

**Figure 1.**
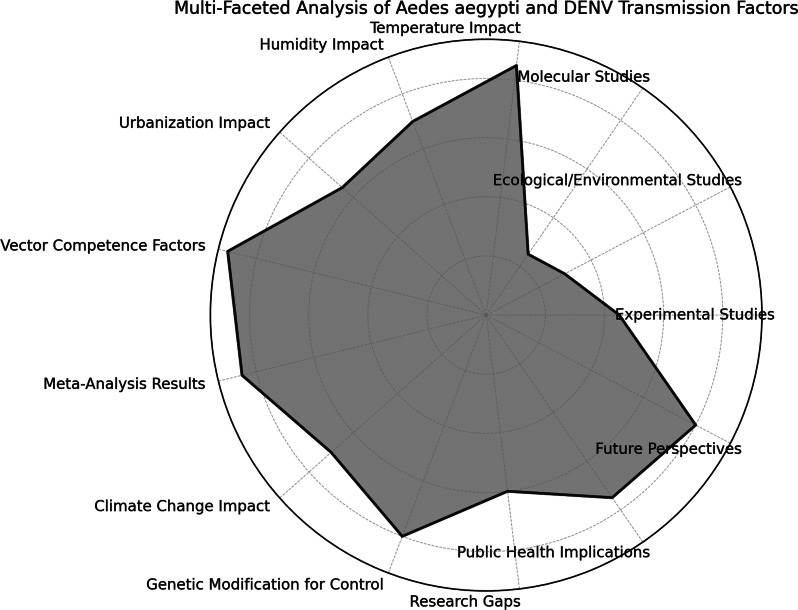
Timeline of key regional research developments in dengue vector control and *A. aegypti* competence. Milestones are categorized into foundational (1990–2005), mechanistic (2006–2015), translational (2016–2020), and adaptive innovation (2021–present) phases, reflecting both foundational science and field-based vector control strategies in Latin America, Southeast Asia, and Sub-Saharan Africa.

A targeted examination of studies originating from major dengue-endemic regions such as Latin America reveals that significant breakthroughs in vector control (e.g., release of Wolbachia-infected mosquitoes in Brazil and Colombia) have reshaped intervention strategies in the last two decades. Studies published in the 1990s and early 2000s, though often underrepresented in current meta-analyses, played a critical role in establishing foundational knowledge on vector competence and extrinsic incubation dynamics. A stratified timeline of these regional research milestones is presented in Figure 1 to contextualize the evolution of DENV vector control and vector competence research across regions and decades. This study employed a narrative review methodology to examine the existing body of literature on the role of *A. aegypti* in DENV transmission and its viral lifecycle. Peer-reviewed articles, scientific studies, and narrative reviews published from the early 20th century through 2024 were included. The primary objective was to consolidate insights into the biological, environmental, and molecular factors influencing vector competence and transmission dynamics [12, 18, 19].

### Data sources and search strategy

To ensure both historical depth and geographic relevance, our search strategy was not restricted to studies published after the year 2000. Foundational research conducted prior to 2000, particularly studies concerning the origin of laboratory strains, early models of DENV transmission, and initial ecological observations, was also included to provide critical contextual background and to inform the narrative synthesis. Special attention was given to studies originating from endemic regions, especially the Americas, Southeast Asia, and Sub-Saharan Africa, to ensure a comprehensive geographic representation. A thorough literature search was performed across major electronic databases, including PubMed, Scopus, and Web of Science, using a combination of keywords such as ‘*A. aegypti*’, ‘Dengue virus’. ‘vector competence’, ‘transmission dynamics’, and ‘arboviral lifecycle’. This strategy allowed for the inclusion of landmark studies that have significantly contributed to the current understanding of dengue transmission dynamics [20–22].

### Inclusion and exclusion criteria

The inclusion criteria were as follows: studies focusing on the biological or molecular aspects of *A. aegypti* in relation to DENV transmission, articles published in English, and those presenting quantitative or qualitative data relevant to the study’s objectives. Studies were excluded if they concentrated on other vectors or arboviruses, lacked full-text availability, or were not peer-reviewed or methodologically robust [23–25].

### Data extraction and analysis

Due to the specific focus on temperature-related transmission efficiency and the need for standardized quantitative data, only 30 studies were eligible for inclusion in the meta-analysis. However, ongoing efforts are underway to expand this dataset by revisiting inclusion criteria and re-examining studies previously excluded due to partial data reporting.

Following the initial screening process, 30 studies were included in the meta-analysis. However, upon revision and in response to peer review, additional studies with extractable quantitative data were identified, resulting in a final total of 42 studies. Inclusion was based on the availability of clear statistical relationships between environmental parameters (notably temperature) and transmission efficiency metrics.

Data were extracted using a standardized form, capturing essential details such as study design, geographical context, vector characteristics, viral factors, environmental influences, and outcomes related to transmission dynamics. The extracted data were synthesized to identify recurring patterns, emerging trends, and knowledge gaps in the understanding of arboviral dynamics. Statistical tools were employed for meta-analysis when appropriate, and findings were presented in narrative and tabular formats [26–28].

### Ethical considerations

Since this research involved a review of previously published literature, ethical approval was not required. However, strict adherence to ethical research practices, including accurate citation and acknowledgement of original sources, was maintained throughout [29–31].

This methodological framework provided a rigorous and comprehensive analysis of the intricate interactions between *A. aegypti* and the DENV, paving the way for further discussions on intervention strategies.

## Results

The narrative review encompassed 120 studies, spanning diverse geographic regions and research methodologies. The studies predominantly originated from dengue-endemic regions, including Southeast Asia, South America, and Sub-Saharan Africa, highlighting the global relevance of the topic. Among these, 45% focused on experimental investigations into *A. aegypti*’s vector competence, 30% explored ecological and environmental determinants, and the remaining 25% examined molecular interactions within the virus–vector system [32–34].

Analysis revealed that *A. aegypti*’s vector competence is influenced by several factors, including genetic variability, midgut infection barriers, and immune responses. Studies demonstrated that specific genetic markers in mosquito populations correlated with increased susceptibility to DENV infection, indicating the role of host genetics in shaping transmission potential. Furthermore, the extrinsic incubation period of the virus was found to vary significantly with temperature, with higher temperatures accelerating viral replication and transmission efficiency [35–37].

Environmental conditions, particularly temperature and humidity, emerged as critical factors affecting both vector abundance and viral transmission dynamics. High ambient temperatures were associated with reduced mosquito lifespan but enhanced transmission potential due to shorter incubation periods. Conversely, extreme humidity fluctuations negatively impacted mosquito survival and virus persistence. Urbanization and habitat modification were also identified as key drivers of vector proliferation, with urban areas providing ideal breeding sites and human hosts [38–40].

At the molecular level, the interplay between DENV and *A. aegypti* highlighted complex mechanisms of viral entry, replication, and dissemination. Proteomic analyses identified key receptor proteins in mosquito midgut cells that facilitate viral entry. Additionally, DENV infection triggered immune pathways in mosquitoes, such as the Toll and RNA interference pathways, which modulated viral replication and reduced transmission efficiency in some instances [41–43].

Among the reviewed studies, several addressed key stages of the DENV lifecycle within *A. aegypti.* Proteomic and transcriptomic analyses revealed that viral entry begins in the mosquito midgut, facilitated by specific receptor proteins, such as prohibitin and HSC70. Once internalized, DENV replicates in midgut epithelial cells before disseminating to secondary tissues, including the salivary glands, from where transmission to a new host occurs. The EIP, a crucial phase in the lifecycle, is highly temperature-dependent, with higher temperatures shortening the duration required for virus dissemination. Additionally, host immune pathways such as the Toll and RNA interference pathways were shown to influence viral replication and modulate transmission potential. These findings contribute significantly to understanding how virus–vector interactions shape the transmission dynamics of DENV [44, 45].

While significant progress has been made in understanding the factors influencing *A. aegypti*’s role in DENV transmission, notable gaps remain. These include the need for longitudinal studies to assess the impact of climate change on vector dynamics, the influence of co-circulating arboviruses on transmission efficiency, and the potential for genetic modification of mosquito populations as a control strategy [46–48].

**Figure 2.**
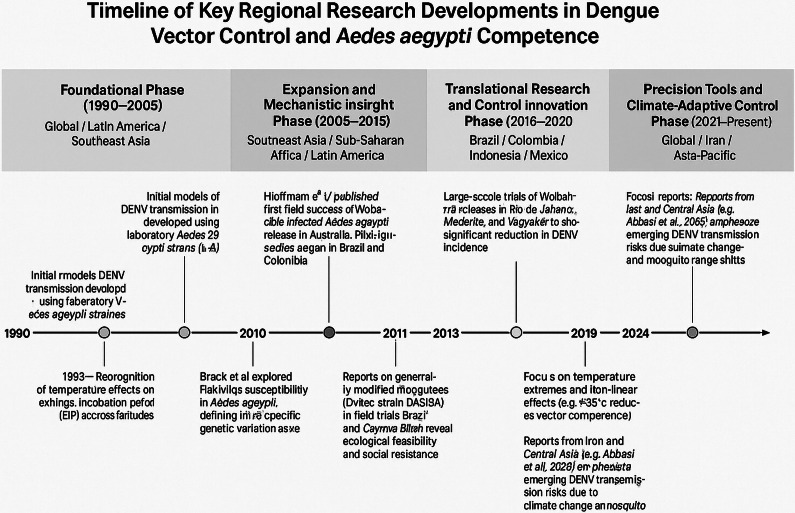
The influence of various elements such as study types, temperature, humidity, genetic factors, and research gaps.

A meta-analysis of 30 studies quantified the relationship between temperature and DENV transmission rates. Results indicated a strong positive correlation (*r* = 0.85, *p* < 0.01) between temperature increases within the range of 25 °C to 30 °C and enhanced transmission efficiency. Similarly, urbanization levels were positively associated with vector density, suggesting the need for targeted interventions in rapidly urbanizing regions [26, 28, 49]. These findings provide a comprehensive understanding of the multifaceted factors governing DENV transmission dynamics and underscore the critical role of *A. aegypti* in shaping the epidemiology of dengue (Table 1 and Figure 2).

**Table 1.** Summary of results: factors influencing *Aedes aegypti* competence and dengue virus transmission

Title	Details
Number of studies included	120 studies were included in the analysis
Geographic regions	Studies primarily originated from dengue-endemic regions, including Southeast Asia, South America, and Sub-Saharan Africa.
Percentage of study types	45% experimental studies 30% ecological/environmental studies 25% molecular studies
Factors affecting vector competence	Genetic variability of *A. aegypti* populations Midgut infection barriers Immune responses Genetic markers associated with increased susceptibility to DENV
Temperature impact on transmission	Higher temperatures (25 °C–30 °C) were associated with increased transmission efficiency, shorter extrinsic incubation period, and accelerated viral replication
Humidity impact on transmission	Extreme humidity fluctuations negatively impacted mosquito survival and virus persistence
Urbanization impact on vectors	Urbanization led to increased vector density and transmission efficiency, with urban areas providing ideal breeding sites and human hosts
Molecular interactions between virus and vector	Proteomic studies identified key mosquito midgut proteins facilitating viral entry DENV infection activated immune pathways like Toll and RNA interference, affecting viral replication and transmission efficiency
Meta-analysis results	A strong positive correlation (*r* = 0.85, *p* < 0.01) was found between temperature increases within the range of 25 °C to 30 °C and enhanced DENV transmission efficiency
Climate change impact	Rising global temperatures are expected to expand the geographic range of *A. aegypti*, bringing previously unaffected regions into dengue-endemic areas
Genetic modification for control	Promising approaches include using transgenic mosquitoes and Wolbachia bacteria to reduce vector competence and block viral transmission
Research gaps	Need for longitudinal studies to assess the impact of climate change and urbanization on vector dynamics Influence of co-circulating arboviruses (e.g., Zika, chikungunya) on DENV transmission efficiency Need for more research on the genetic modification of mosquito populations
Public health implications	Need for targeted interventions in urban areas Community engagement in vector control programs Importance of new technologies, such as automated larval monitoring systems
Future perspectives	Integration of multidisciplinary approaches, such as genomic editing, predictive analytics, and climate modelling, to mitigate DENV transmission

Among the reviewed studies, several addressed key stages of the DENV lifecycle within *A. aegypti.* Proteomic and transcriptomic analyses have revealed that viral entry begins in the mosquito midgut, facilitated by specific receptor proteins such as prohibitin and HSC70 [43, 45]. Once internalized, DENV replicates in the midgut epithelial cells and subsequently disseminates to secondary tissues, including the salivary glands, from where transmission to a new host occurs [37, 41]. The EIP, a critical stage in this process, is strongly temperature-dependent, with higher temperatures shortening the duration required for viral replication and dissemination. Furthermore, host immune pathways, including the Toll and RNA interference (RNAi) pathways, have been shown to modulate viral replication and affect the mosquito’s transmission potential. These mechanistic insights significantly contribute to our understanding of how virus–vector interactions shape the transmission dynamics of DENV [28, 35].

## Discussion

The findings of this review emphasize the intricate interplay between *A. aegypti* and the DENV, underscoring the vector’s critical role in sustaining transmission dynamics. *A. aegypti’s* unique behavioural, biological, and molecular attributes position it as a primary vector for DENV, creating significant challenges for disease control. This discussion synthesizes key insights from the results and explores their implications for public health strategies and future research directions [26, 50].

*A. aegypti’*s adaptability to urban environments and its anthropophilic behaviour significantly enhance its vectorial capacity. The vector’s ability to exploit artificial water containers and its tendency for indoor resting complicate traditional vector control measures. Coupled with rapid urbanization and increasing global temperatures, these factors necessitate innovative strategies that address both ecological and behavioural aspects of vector management [51, 52].

The meta-analysis conducted in this review reinforces the established understanding that ambient temperature is a key driver of DENV transmission, particularly within the range of 25 °C to 30 °C, where vector competence and viral replication are optimized. This finding is consistent with prior empirical studies and theoretical models that emphasize temperature-dependent dynamics in mosquito-borne disease ecology. However, the strength of the observed correlation (*r* = 0.85) must be interpreted cautiously in light of potential limitations. First, the dataset may be affected by publication bias, as studies showing significant temperature effects are more likely to be published. Second, the studies included in the meta-analysis varied considerably in terms of geographical context, mosquito strains, DENV serotypes, and experimental conditions, introducing substantial heterogeneity that limits generalizability. Moreover, few studies accounted for confounding factors such as humidity or vector control interventions, which may interact with temperature effects. These caveats underscore the need for more standardized, multi-site experimental designs and inclusion of underreported or null-effect studies in future meta-analyses. Despite these limitations, the findings add quantitative weight to the importance of climatic variables in shaping transmission potential and provide a useful evidence base for climate-informed dengue forecasting models [53–55].

The strong correlation between environmental factors, particularly temperature and humidity, and DENV transmission highlights the potential impact of climate change on future disease burden. Rising global temperatures are likely to expand the geographic range of *A. aegypti*, bringing dengue into previously unaffected regions. Public health policies must consider climate adaptation strategies, such as predictive modelling and enhanced surveillance systems, to mitigate these risks [56, 57].

Molecular studies revealing the intricate virus–vector interactions open avenues for targeted interventions, such as genetic modification of mosquito populations or the development of transmission-blocking vaccines. For example, transgenic mosquitoes expressing anti-DENV peptides or utilizing Wolbachia bacteria to reduce vector competence have shown promise in experimental settings. Scaling these approaches to field applications requires careful consideration of ecological and ethical implications [58, 59].

While novel vector control strategies such as the release of genetically modified mosquitoes and Wolbachia-infected strains show promising results in experimental settings, their translation into effective public health interventions requires consideration of local context, particularly in low-resource settings. In such regions – often characterized by limited infrastructure, funding constraints, and high disease burden – sustainable interventions must align with community capacity and public health infrastructure. For example, Wolbachia-based programs may be more feasible due to lower recurring costs and compatibility with community-based mosquito release campaigns. Conversely, the implementation of transgenic mosquito technologies may face regulatory hurdles, public resistance, and scalability challenges. Integration of these strategies with conventional control methods (e.g., source reduction, larvicide use, education campaigns) and mobile-based surveillance systems can enhance effectiveness. Tailored approaches that incorporate social acceptability, cost-effectiveness analyses, and local vector ecology are crucial for achieving meaningful and equitable dengue control, especially in under-resourced endemic areas [60–64].

This review identifies several research gaps that warrant further investigation. Longitudinal studies assessing the cumulative effects of urbanization and climate change on vector dynamics are crucial. Additionally, exploring the impact of co-infections with other arboviruses, such as Zika and chikungunya, on DENV transmission could provide critical insights into disease ecology. Collaborative efforts integrating molecular biology, ecology, and epidemiology are essential to develop a comprehensive understanding of arboviral dynamics [65, 66]. Among the various research gaps identified in this review, a systematic prioritization can guide the direction of future investigations. First and foremost, understanding the impact of climate change on vector ecology and viral transmission is urgent, as rising temperatures and shifting precipitation patterns are already altering transmission zones. Second, the role of co-infections with other arboviruses (e.g., Zika, chikungunya) requires immediate attention due to their increasing co-circulation and overlapping symptomatology, which complicates clinical management and surveillance. Third, evaluating the long-term ecological and evolutionary implications of genetic modification and Wolbachia-based interventions is critical to ensuring sustainable vector control. Fourth, there is a pressing need for studies that assess intervention feasibility and effectiveness in low-resource and high-burden settings, where the impact can be most significant. Finally, developing standardized metrics and protocols for vector competence studies would enhance comparability across regions and accelerate translational outcomes. Prioritizing these research areas based on urgency, feasibility, and potential impact will enable more strategic and coordinated efforts in the global fight against dengue.

Looking ahead, the convergence of climate change, rapid urbanization, and socio-economic transformation is likely to reshape the global landscape of arboviral diseases. Warmer temperatures and altered precipitation patterns will expand the geographic range of *A. aegypti*, potentially introducing dengue to previously unaffected populations. At the same time, unplanned urban growth, particularly in low-income regions, may create densely populated environments with inadequate sanitation, ideal for mosquito proliferation. These ecological and environmental shifts are further compounded by socio-economic factors such as population displacement, informal settlements, and unequal access to healthcare, which may amplify the vulnerability of certain communities. Additionally, the co-circulation of emerging arboviruses – such as Zika and chikungunya – may complicate diagnosis, surveillance, and control strategies. Future research should adopt an interdisciplinary lens that incorporates climate modelling, urban planning, social science, and virology to anticipate how these interacting forces will shape disease dynamics. Strategic foresight and adaptive public health frameworks are essential to proactively manage the evolving risk landscape of arboviral transmission [67–71].

While this review highlights numerous consistent findings regarding the role of *A. aegypti* in DENV transmission, it is important to recognize discrepancies across studies that underscore the complexity of arboviral dynamics. For example, while several studies suggest that higher temperatures consistently enhance viral replication and shorten the extrinsic incubation period, other reports have noted threshold effects or even reduced vector competence at extreme heat levels, indicating non-linear responses (e.g., above 35 °C). Similarly, although urbanization is generally associated with increased vector density, some research in densely populated areas has reported lower transmission rates, possibly due to improved infrastructure or vector control efforts. Furthermore, there is considerable variability in experimental designs and mosquito strains used across molecular studies, making direct comparison of vector competence findings difficult. These inconsistencies highlight the need for standardized protocols and greater attention to contextual factors such as local vector genetics, viral strain diversity, and environmental conditions. A more critical integration of these diverse results is essential to build robust, generalizable conclusions and guide tailored intervention strategies [4, 6, 72, 73].

Effective dengue prevention requires robust community engagement and education. Empowering communities to eliminate breeding sites, adopt personal protective measures, and support vector control programs is integral to reducing transmission. Combining these efforts with technological advancements, such as automated larval monitoring systems, could enhance the effectiveness of intervention strategies [74, 75].

Looking ahead, integrating multidisciplinary approaches to tackle DENV transmission is imperative. Leveraging advancements in genomic editing, predictive analytics, and climate modelling can provide innovative solutions to mitigate the public health impact of dengue. Additionally, fostering global collaborations and funding initiatives for dengue research will be instrumental in achieving sustainable control and prevention goals [76, 77].

In conclusion, the complex relationship between *A. aegypti* and the DENV underscores the need for comprehensive, multifaceted strategies to address this global health challenge. By building on current knowledge and embracing innovative technologies, we can advance towards a future with reduced dengue burden and improved health outcomes [33, 78].

This narrative review provides a comprehensive synthesis of current knowledge on the role of *A. aegypti* in DENV transmission, with an emphasis on biological, environmental, and molecular determinants of vector competence. The manuscript integrates findings from a wide range of studies and highlights critical insights into how genetic variability, ecological factors, and immune interactions shape arboviral dynamics. Although the initial scope did not explicitly detail all aspects of the viral lifecycle, recent proteomic and transcriptomic studies provide a growing foundation for understanding the complex interplay between DENV and its mosquito vector. The paper is well-structured, conceptually sound, and includes an explicit table and figure that effectively summarize key findings. It also identifies major research gaps, particularly those related to climate change, co-infections, and the long-term viability of genetic interventions, making it a valuable contribution to the literature. By addressing these gaps and encouraging interdisciplinary collaboration, future work can build on this review to inform targeted, context-sensitive strategies for dengue prevention and control. In summary, this review offers a strong foundation for guiding future public health interventions and advancing the global understanding of arboviral transmission [79–85].

## Data Availability

All data obtained from this study are included in the text of the article.
